# Modeling study of the heat of absorption and solid precipitation for CO_2_ capture by chilled ammonia

**DOI:** 10.1039/c9ra00164f

**Published:** 2019-06-27

**Authors:** Qiang Zhou, Lan Liu, Eric Croiset, Zhongchao Tan, Qingcai Liu, Jian Yang

**Affiliations:** College of Materials Science & Engineering, Chongqing University Chongqing China skyinjune@cqu.edu.cn; Faculty of Engineering, University of Waterloo 200 University Avenue West Waterloo Ontario Canada N2L 3G1

## Abstract

The contribution of individual reactions to the overall heat of CO_2_ absorption, as well as conditions for solid NH_4_HCO_3_(s) formation in a chilled ammonia process (CAP) were studied using Aspen Plus at temperatures between 2 and 40 °C. The overall heat of absorption in the CAP first decreased and then increased with increasing CO_2_ loading. The increase in overall heat of absorption at high CO_2_ loading was found to be caused mostly by the prominent heat release from the formation of NH_4_HCO_3_(s). It was found that NH_4_HCO_3_(s) precipitation was promoted for conditions of CO_2_ loading above 0.7 mol CO_2_/mol NH_3_ and temperatures less than 20 °C, which at the same time can dramatically increase the heat of CO_2_ absorption. As such, the CO_2_ loading is recommended to be around 0.6–0.7 mol CO_2_/mol NH_3_ at temperatures below 20 °C, so that the overall absorption heat is at a low state (less than 60 kJ mol^−1^ CO_2_). It was also found that the overall heat of CO_2_ absorption did not change much with temperature when CO_2_ loading was less than 0.5 mol CO_2_/mol NH_3_, while, when the CO_2_ loading exceeded 0.7 mol CO_2_/mol NH_3_, the heat of absorption increased with decreasing temperature.

## Abstract 2

Prediction of (a) solution speciation change and (b) heat of CO_2_ absorption in the chilled ammonia process (CAP).

## Graphical abstract

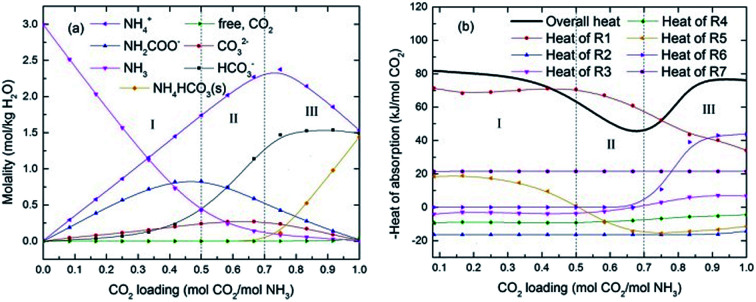

## 1. Introduction

CO_2_ is considered as the main greenhouse gas responsible for global warming and climate change.^1^ According to the Intergovernmental Panel on Climate Change (IPCC), carbon capture and storage (CCS) is an attractive technology for reduction of greenhouse gas emissions in the medium term.^2^ There are three main types of carbon capture technology: pre-combustion, oxy-combustion, and post-combustion.^3–6^

Post-combustion capture attracts the most attention because it can be more easily implemented on existing power plants.^7–9^ In post-combustion capture, alkanolamine solutions, monoethanolamine (MEA) in particular, act as CO_2_ absorbents with high reaction rates.^10–12^ However, amine-based capture suffers from corrosion and high operating cost, including absorbent degradation and relatively high energy consumption. These drawbacks greatly hinder its wide deployment in the electric power industry.^13–16^ Many researchers investigated cost-effective alternatives with low heat of CO_2_ absorption. Aqueous ammonia (NH_3_) is considered as a competitive candidate because of its unique properties, including (1) high CO_2_ capture capacity;^17^ (2) simultaneous capture of multiple acidic gases such as SO_2_ and NO_*x*_;^18,19^ (3) resistance to oxidation and thermal stability;^10^ (4) low capital costs; (5) relatively low heat of CO_2_ absorption. The heat of CO_2_ absorption by aqueous NH_3_ at 40 °C has been experimentally measured and reported by Liu *et al.*^20^ and Qin *et al.*^21^ (around 65–70 kJ mol^−1^ CO_2_), which is lower than that of the MEA system reported by Kim *et al.*^14^ (more than 80 kJ mol^−1^ CO_2_ at 40 °C).

In view of the fact that ammonia escape appears to be the greatest concern to the industry, the chilled ammonia process (CAP) has being developed to address this problem.^22^ In a CAP process, CO_2_ is absorbed at low temperatures in the range of 2–20 °C to minimize the volatilization of ammonia. The CO_2_-enriched solution is then regenerated at 100–150 °C and 2–136 atm. Bak *et al.*^23^ pointed out that, when the absorber operated at a feed gas temperature of 10 °C and lean solution at a temperature of 7 °C, the CO_2_ absorption efficiency could reach more than 85% with ammonia loss less than 8%.

However, there is limited information on the contribution of each individual reaction occurring during CO_2_ absorption by NH_3_ to the overall heat of CO_2_ absorption in CAP. In addition, conditions for the formation of solid ammonium bicarbonate, NH_4_HCO_3_(s), must be well understood. Since the temperatures in CAP are low in general, solid may precipitate in the absorber. Yu *et al.* analyzed the solid composition in the absorber by XRD, the result suggested that the pilot plant samples were predominantly NH_4_HCO_3_(s).^24^ Besides, Diao *et al.* studied the crystalline solids by FT-IR analysis, the FT-IR patterns of the crystalline solids were compared to standard ammonium bicarbonate powders. They found that ammonium bicarbonate was the main product.^25^ NH_4_HCO_3_(s) formation would dramatically change the heat of CO_2_ absorption of the NH_3_–CO_2_–H_2_O system, because of the exothermic property of NH_4_HCO_3_(s) formation.^26^ The heat of CO_2_ absorption is an important thermodynamic property, as a higher heat of CO_2_ absorption means more energy required in solvent regeneration. The detailed thermodynamic analysis for the contribution of each individual reaction to the overall heat of absorption is one of the key ways to clarify the reaction mechanism and process optimization. According to the exothermic/endothermic characteristics of each individual reaction, the operating parameters such as CO_2_ loading and temperature, can be adjusted to optimize system energy consumption. Therefore, some researchers studied the heat of absorption for each individual reaction in amine-based capture system^27^ and ammonia-based system,^28^ but temperatures ranged from 40 to 80 °C, which were much higher than those encountered in CAP; in addition, at those higher temperatures solid precipitation was not observed and not considered an issue. Energy consumption in CAP has been evaluated by thermodynamic models,^29,30^ but they all focused on the whole process rather than analyzed the heat change caused by each individual chemical reaction in the absorber. Although Jilvero *et al.*^31^ and Kurz *et al.*^32^ reported phase equilibrium experimental data for the NH_3_–CO_2_–H_2_O system at temperatures in the range 10–80 °C, the effect of solid formation on heat of absorption was not reported in their studies. The contribution of each individual reaction to the overall heat of CO_2_ absorption in CAP is a gap, which is very important to understand the absorption mechanism and control the system absorption heat. The various contributions can be controlled by adjusting the operation parameters, such as CO_2_ loading and temperature, to optimize overall heat of absorption.

In this work, the heat of CO_2_ absorption and the contribution of each individual reaction, particularly that of NH_4_HCO_3_(s) formation, to the overall heat of CO_2_ absorption in CAP is investigated through a thermodynamic model. The model is first validated by experimental data from literature, and then the validated model is used to predict the heat of absorption in CAP. Finally, according to NH_4_HCO_3_(s) formation conditions, recommended CO_2_ loading at different temperatures with the lowest overall heat of absorption are proposed.

## 2. Methodology

It is difficult to experimentally determine each individual reaction's contribution to the overall heat of CO_2_ absorption. Thermodynamic analysis is proved to be a useful and powerful method to study the absorption process and absorption heat in CO_2_ capture systems.^27–29^ Two models that are commonly used in thermodynamics studies of CO_2_ capture process: (1) the extended UNIQUAC model developed by Thomsen and Rasmussen^33^ and (2) the e-NRTL model proposed by Chen *et al.*^34^ Gudjonsdottir *et al.*^35^ reported that, if the interaction parameters better fit the experimental data in the NH_3_–CO_2_–H_2_O system, the e-NRTL model covers a wider range of conditions than the extended UNIQUAC model. Jilvero *et al.*^31^ also demonstrated that the e-NRTL model is more accurate for the prediction of CO_2_ partial pressure at low temperatures (10–40 °C).

There are two commonly ways for calculating absorption heat. The van't Hoff equation based on equilibrium constant (eqn (3))^27,28^ and a thermodynamic relation based on VLE data (eqn (6)).^36,37^ The van't Hoff equation (eqn (3)) is derived directly from the general form of Gibbs–Helmholtz equation (G–H equation),^37^ and the general form of G–H equation is:^38^1
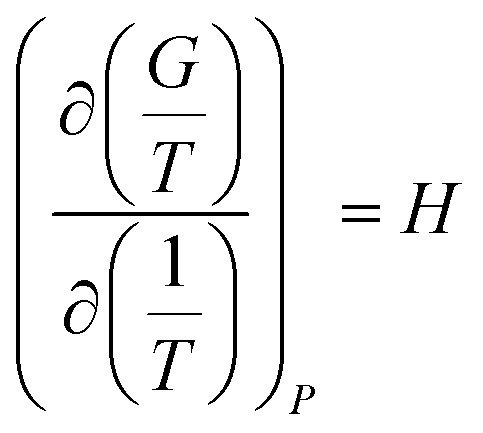


Further, the relationship between the equilibrium constant and Gibbs free energy is:

2 Δ*G* = −*RT* ln *K*

Eqn (2) can be substituted into eqn (1) and we can obtain the van't Hoff equation:3
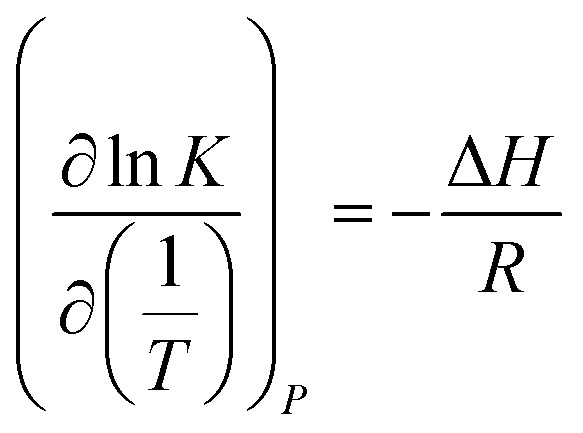


For the thermodynamic relation based on VLE data (eqn (6)), Sherwood and Prausnitz (1962) gave a detailed description in their paper. The general expression for calculating the absorption heat is:^39^4
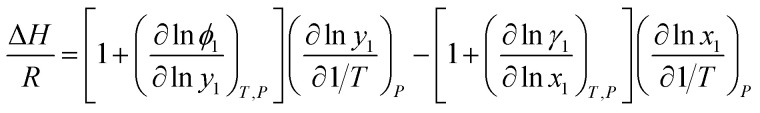
where, *ϕ* is vapor phase fugacity coefficient, *y* is mole fraction in vapor phase, *γ* is liquid phase activity coefficient and *x* is mole fraction in liquid phase, subscripts 1 is lighter component.

Eqn (4) is perfectly general, as no simplifying physical assumptions have been made. However its application in this form requires extensive data in the single-phase vapor and liquid regions. Sherwood and Prausnitz point out that eqn (4) can be simplified to eqn (5) after some simplifying physical assumptions.^39^5
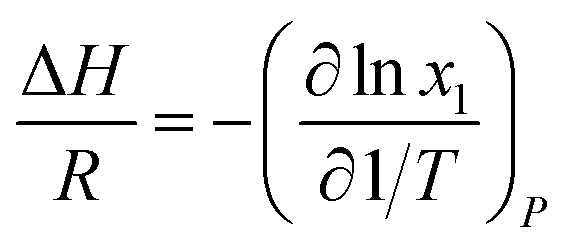


For simplification at ambient pressures, CO_2_ partial pressures are always used instead of CO_2_ solubility in eqn (5) that the absorption heat can be obtained simply from VLE data.^36,37^6
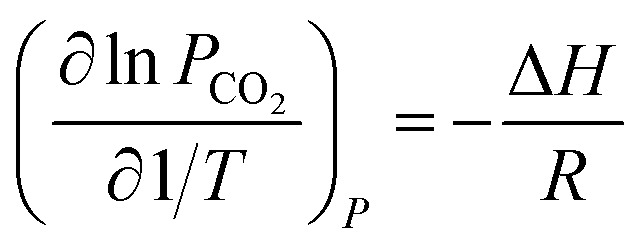


**Fig. 1:**
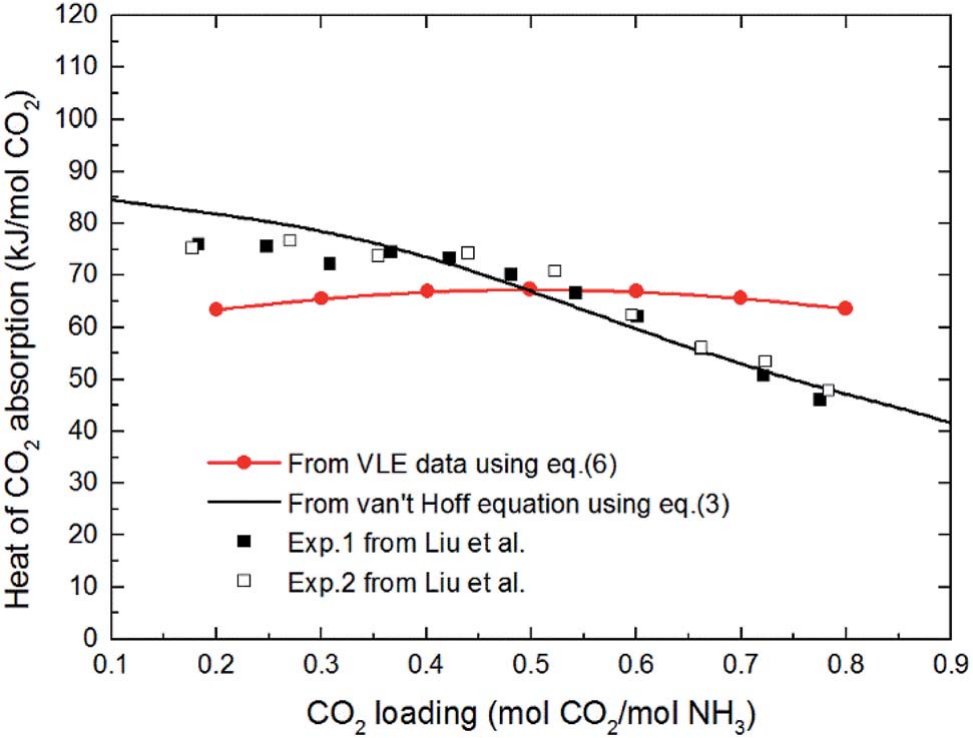
The comparison of difference between the absorption heat calculated by the two methods and the experimental data reported by Liu *et al.* at 40 °C (VLE data in eqn (6) from Kurz *et al.* 1995 (ref. 32)).

The comparison of difference between the absorption heat calculated by the above two methods and the experimental data reported by Liu *et al.*^20^ is illustrated in Fig. 1. It clearly shows that the values for CO_2_ absorption heat calculated by van't Hoff equation based on equilibrium constant (eqn (3)) agree better with experimental data than that by thermodynamic relation based on VLE data (eqn (6)). The main reason is that van't Hoff equation based on equilibrium constant (eqn (3)) is derived directly from the general form of G–H equation, as no assumptions have been made; however, the use of thermodynamic relation based on VLE data (eqn (6)) implies inherent assumptions,^37,39,40^ which reduces the accuracy of eqn (6). Additionally, thermodynamic relation based on VLE data (eqn (6)) can only give us the overall absorption heat, but the current study mainly focuses on the endothermic/exothermic condition of each individual reaction. Therefore, in this paper, the van't Hoff equation based on equilibrium constant is selected to calculate the heat of each reaction.

According to the above description, in this study e-NRTL model integrated in Aspen Plus is used to describe the liquid phase activity coefficients. The van't Hoff equation based on equilibrium constant is selected to calculate the heat of each reaction. The flash module in Aspen Plus (V7.2) is chosen to calculate the chemical equilibrium and solution speciation. Then the heat of CO_2_ absorption can be obtained from the solution speciation and chemical equilibrium constants.

### 2.1 Chemical equilibrium

The chilled NH_3_–CO_2_–H_2_O system herein comprises the following species: CO_2_, NH_3_, H_2_O, NH_4_^+^, HCO_3_^−^, CO_3_^2−^, NH_2_COO^−^, H_3_O^+^, OH^−^, and solid precipitates (NH_4_HCO_3_(s)). The solid NH_4_HCO_3_(s) is assumed to be the only solid species in the solution.^24,25,35^ The main reactions taking place in this system are as follows:R1
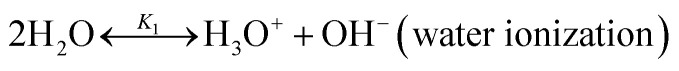
R2
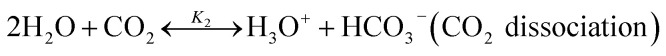
R3
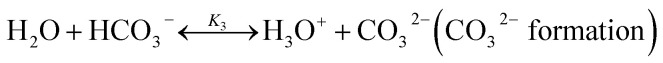
R4
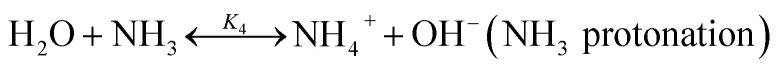
R5
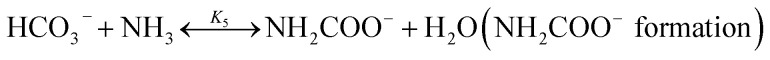


In CAP, the formation of NH_4_HCO_3_(s) is described byR6
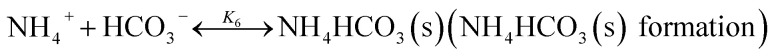


In addition, CO_2_ dissolution should be considered, that is,R7
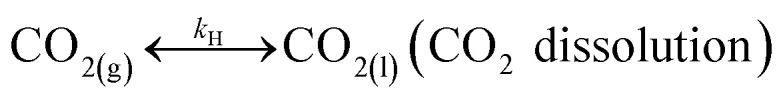


The chemical equilibrium constants *K*_1_–*K*_6_ and the Henry's law constant *k*_H_ can be calculated using eqn (7)^27,41–43^7
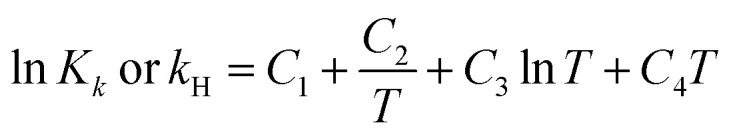
where, *K* is the chemical equilibrium constant of (R1)–(R6); subscript *k* is reaction number, and *k*_H_ is Henry's law constant of (R7). The *C*_1_, *C*_2_, *C*_3_ and *C*_4_ in eqn (7) are parameters that need to select from literature or Aspen Plus databank, and will be explained in the following sections.

N_2_, NH_3_ and CO_2_ are chosen as Henry components in this model. Other acid gases, such as H_2_S, NO_*x*_ and SO_2_ and so on, reduce the overall heat of CO_2_ absorption by aqueous NH_3_ according to Qi *et al.*^28^ results at temperatures more than 40 °C. But the effect of these acid gases on the overall heat of CO_2_ absorption in CAP has not reported in the open literature, these studies will be one of our future works. In this study, we just focus on the chilled NH_3_–CO_2_–H_2_O system, the other impurity acid gases are thus neglected to simplify the model. The default values in Aspen Plus (V7.2) databank are used for parameters of binary interaction and electrolyte pair in the NH_3_–CO_2_–H_2_O system.^32,44–46^

### 2.2 Model of heat of absorption

The heat of each individual reaction ((R1)–(R7)) is expressed in terms of enthalpy change, Δ*H*_*k*_, which can be calculated from the van't Hoff's equation^47^ with corresponding equilibrium constant written as in eqn (8). The results are summarized in Table 1 (the values of *C*_2_ to *C*_4_ will be discussed later).8
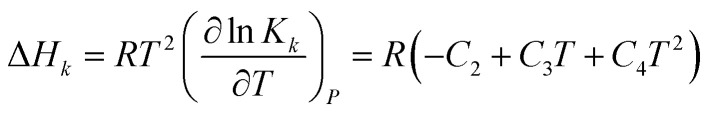


**Table 1:** Enthalpy change (kJ mol^−1^) of reactions (R1)–(R7) calculated using eqn (8) at temperatures between 2 and 40 °C

Reaction no.	Enthalpy change (Δ*H*_*k*_, kJ mol^−1^)						
	2 °C	5 °C	10 °C	15 °C	20 °C	30 °C	40 °C
(R1)	60.37	59.81	58.88	57.94	57.01	55.13	53.27
(R2)	16.39	15.48	13.95	12.42	10.89	7.83	4.77
(R3)	22.19	21.30	19.83	18.35	16.88	13.93	10.98
(R4)	7.83	7.36	6.56	5.74	4.91	3.19	1.42
(R5)	−24.11	−24.11	−24.11	−24.11	−24.11	−24.11	−24.11
(R6)	−23.72	−22.39	−20.21	−18.04	−15.91	−11.70	−7.63
(R7)	−21.51	−21.11	−20.46	−19.79	−19.13	−18.56	−16.43

The overall heat of CO_2_ absorption in the NH_3_–CO_2_–H_2_O system depends on the endothermic or exothermic properties, as well as the extent and direction, of each individual reaction (R1)–(R7) at different CO_2_ loadings. The extent and direction of (R1) to (R7) are determined by the key species change in the solution with changing CO_2_ loading. By increasing the CO_2_ loading gradually, all of these reactions will move in one direction or the other. Some may move forward and the others backward, depending on the variation of key species, Δ*n*_i_, as shown in the following equations:9
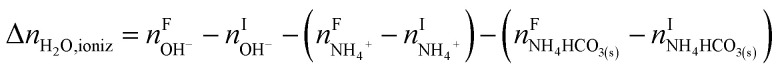
10
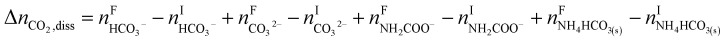
11
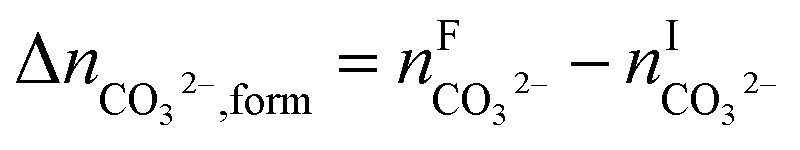
12
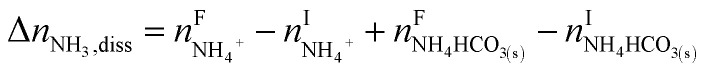
13
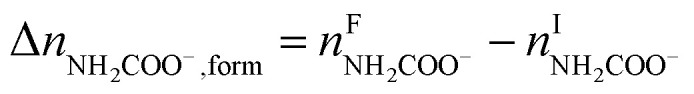
14
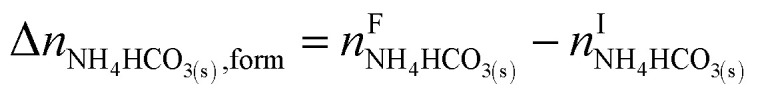


The change in the total number of moles of CO_2_, Δ*n*_CO_2_,tot_ is determined by15
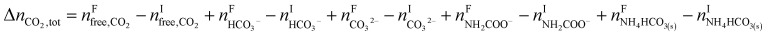
where superscripts F and I stand for final and initial states, respectively.

The extent and direction of each individual reaction absorbing per unit CO_2_ can be quantified by *E*_*k*_:16
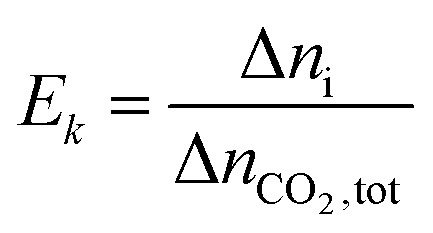
where Δ*n*_i_ is the increment of key species in mole, *E*_*k*_ is the specific extent for each reaction ((R1)–(R7)), *i.e.* per mole of CO_2_ absorbed. *E*_*k*_ value can be positive or negative depending on the direction of the reaction.

The overall heat of CO_2_ absorption can be calculated by the summation of the heat of absorption of all the reactions:17
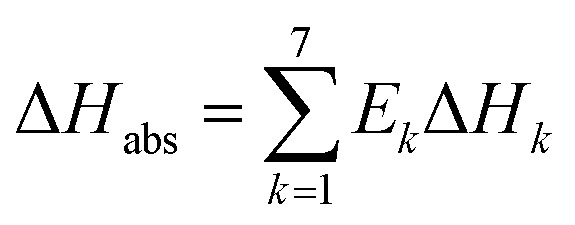
where Δ*H*_abs_ is the overall heat of CO_2_ absorption.

### 2.3 Chemical equilibrium constants

In order to accurately predict the enthalpy change of each reaction, it is important to obtain accurate chemical equilibrium constants. According to eqn (8), the enthalpy change for each individual reaction ((R1)–(R7)) is directly related to the equilibrium constant. The chemical equilibrium constants can be found on mole fraction basis and/or molality basis. In this paper mole fraction basis is used. However, some equilibrium constants available in literature are on molality basis. In this case, unit conversion is done using eqn (18)

18 ln *K*_m_ = ln *K*_*x*_ + Δ*n* ln(55.51)

where *K*_*m*_ is the molality based equilibrium constant; *K*_*x*_ is the mole fraction based equilibrium constant; Δ*n* is the change in moles across the equation excluding water and solid. In this study, the protonation of NH_3_(R4) is taken as an example to explain the choice of the equilibrium constants. The similar method is applied for the other reactions. The equilibrium constants available in literature are listed in Table 2.

**Table 2:** References for choosing chemical equilibrium constants of reactions (R1)–(R7)

Reaction no.	Parameter	References
(R1)	*K*_1_	Austgen *et al.*,^48^ Weiland *et al.*,^41^ Pazuki *et al.*,^49^ Beutier and Renon^50^
(R2)	*K*_2_	Austgen *et al.*,^48^ Pazuki *et al.*,^49^ Beutier and Renon,^50^ Oscarson *et al.*^51^
(R3)	*K*_3_	Austgen *et al.*,^48^ Oscarson *et al.*,^51^ Weiland *et al.*^41^
(R4)	*K*_4_	Edwards *et al.*,^52^ Kawazuishi and Prausnitz,^53^ Clegg and Brimblecombe,^54^ Pazuki *et al.*,^49^ Aspen Plus
(R5)	*K*_5_	Edwards *et al.*,^52^ Kawazuishi and Prausnitz,^53^ Pazuki *et al.*,^49^ Beutier and Renon,^50^ Aspen Plus
(R6)	*K*_6_	Aspen Plus
(R7)	*k*_H_	Austgen *et al.*,^48^ Oscarson *et al.*,^51^ Que and Chen,^55^ Kawazuishi and Prausnitz,^53^ Pazuki *et al.*^49^

#### 2.3.1 Chemical equilibrium constant for NH_3_ protonation (R4)

**Fig. 2:**
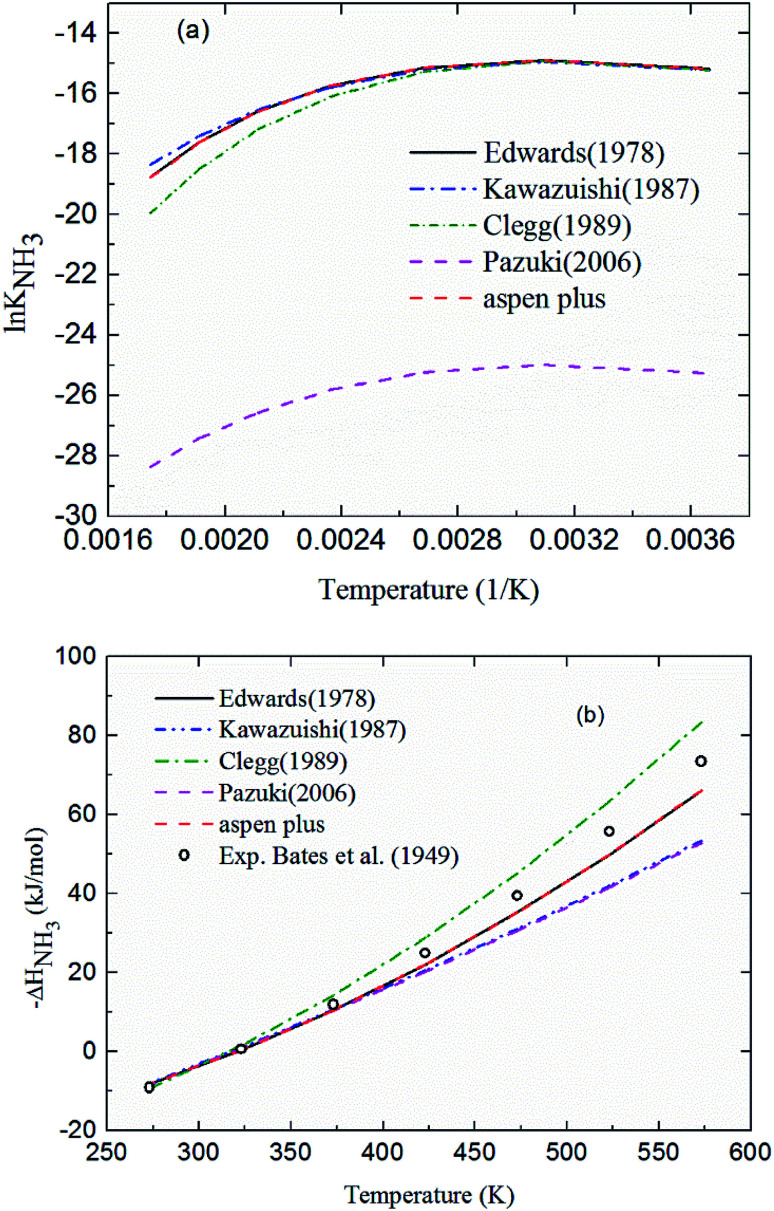
(a) ln *K*_4_ and (b) corresponding −Δ*H*_NH_3__ as a function of temperature for NH_3_ protonation in the water (R4).

Comparing the chemical equilibrium constants from different sources, the one given by Edwards *et al.*^52^ is chosen for NH_3_ protonation (R4) in the current study. Fig. 2(a) shows the equilibrium constants for NH_3_ protonation (R4), in which ln *K*_4_ is given by Edwards *et al.*,^52^ Kawazuishi and Prausnitz,^53^ Pazuki *et al.*,^49^ Clegg and Brimblecombe,^54^ and Aspen Plus (V7.2). The corresponding enthalpy change, −Δ*H*_NH_3__, calculated by eqn (8) are shown in Fig. 2(b) and compared with the experimental data reported by Bates and Pinching.^56^ All equilibrium constants has similar values and tendency except that reported by Pazuki *et al.*^49^ at different temperatures. In Fig. 2(b), the corresponding enthalpy change calculated by Edwards *et al.*^52^ and Aspen Plus (V7.2) have the same values. The enthalpy change calculated by Kawazuishi and Prausnitz^53^ and Pazuki *et al.*^49^ have similar values as well. However, the enthalpy change predicted by Clegg and Brimblecombe^54^ has little difference with the others'. Besides, the prediction of enthalpy change by Edwards *et al.*^52^ is the closest to the experimental data. It should be noted that Edwards *et al.*^52^ and Aspen Plus predict the same values. The black solid line overlaps with the red dotted line in Fig. 2; therefore, only four curves are seen in Fig. 2. The similar method is applied to other reactions. The default equilibrium constant from Aspen Plus (V7.2) databank is used for NH_4_HCO_3_(s) formation (R6). The constants *C*_1_, *C*_2_, *C*_3_ and *C*_4_ for each reaction are summarized in Table 3. One may notice that the values of the parameters for the CO_3_^2−^(R3), NH_3_(R4) and NH_2_COO^−^ formation (R5) in this paper are different from those in the original references, because they are converted using eqn (18) to mole fraction basis.

**Table 3:** Chemical equilibrium constants and Henry's constant for reactions (R1)–(R7)

Reaction no.	Parameters	*C*_1_	*C*_2_	*C*_3_	*C*_4_	Sources
(R1)	*K*_1_	132.90	−13445.90	−22.48	0.00	Austgen *et al.*^48^^a^
(R2)	*K*_2_	231.47	−12092.10	−36.78	0.00	Austgen *et al.*^48^^a^
(R3)	*K*_3_	216.05	−12431.70	−35.48	0.00	Oscarson *et al.*^51^^b^
(R4)	*K*_4_	−1.26	−3335.70	1.50	−0.03706	Edwards *et al.*^52^^b^
(R5)	*K*_5_	−4.58	2900.00	0.00	0.00	Edwards *et al.*^52^^b^
(R6)	*K*_6_	554.82	−22442.53	−89.01	0.06473	Aspen Plus^a^
(R7)	*k*_H_	170.71	−8477.71	−21.96	0.00578	Aspen Plus^a^

a Mole fraction based chemical equilibrium constants in references mentioned.

b Molality based equilibrium constants in references mentioned.

## 3. Results and discussion

### 3.1 Model validation

The model validation is conducted by comparing the model results with experimental data obtained from literature. The calculation results are obtained for vapor–liquid equilibrium (VLE), solid–liquid equilibrium (SLE), and solution speciation at different temperatures and NH_3_ concentrations. They are introduced as follows.

#### 3.1.1 Validation of the thermodynamic model in vapor phase (VLE)

**Fig. 3:**
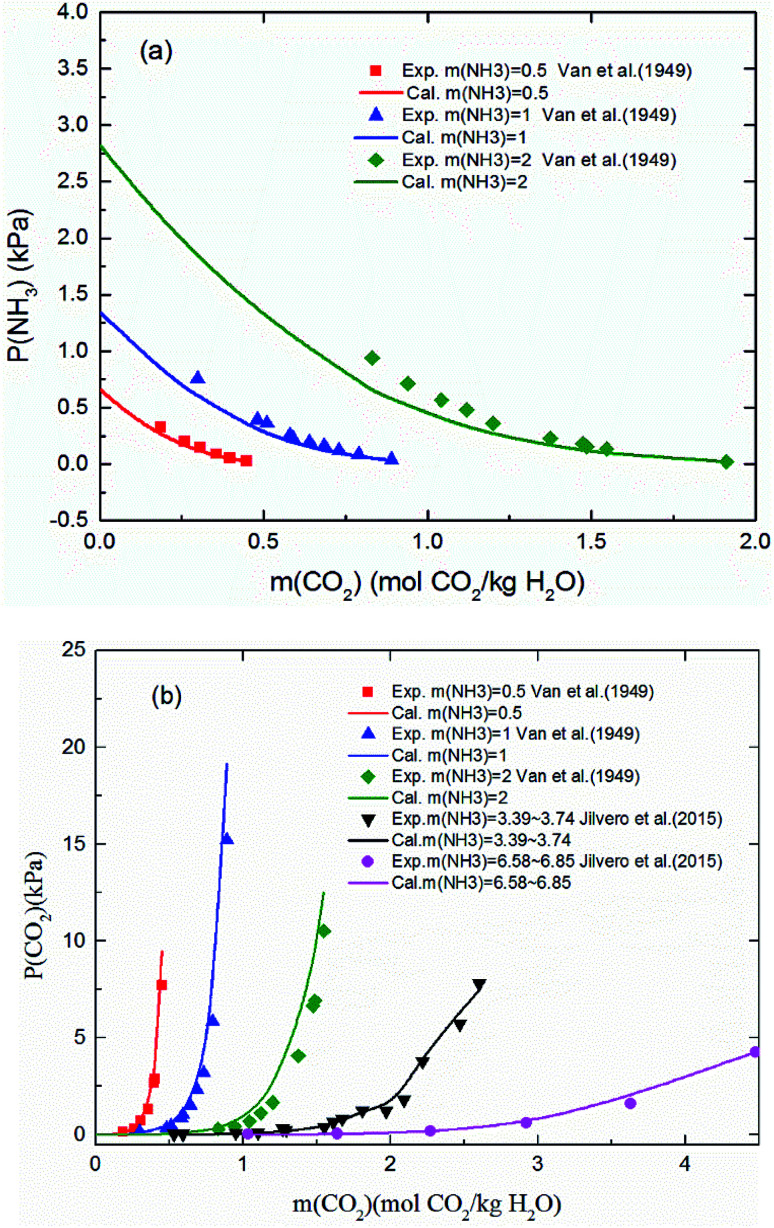
Comparison of the calculated (a) NH_3_ and (b) CO_2_ equilibrium partial pressure with experimental data^31,57^ at 20 °C.

Fig. 3 shows the predicted NH_3_ and CO_2_ partial pressure at *T* = 20 °C and different NH_3_ molality. The model is in good agreement with the experimental data from different laboratories, which indicates the reliability of the model results.^31,57^ There is no NH_3_ equilibrium partial pressure reported in Jilvero's article. Therefore, only the CO_2_ equilibrium partial pressure is exhibited in Fig. 3(b). With increasing CO_2_ molality, the equilibrium partial pressure of NH_3_ decreases. Because free NH_3_ in solution is consumed to form nitrogenous compounds at a higher CO_2_ molality, it lowered the mass transfer driving force for ammonia escaping. Therefore, a high CO_2_ molality is recommended in order to reduce, not only ammonia escape^58^ but also the regeneration energy consumption.^59^ It can be observed that at low NH_3_ concentration (less than 1 mol NH_3_/kg H_2_O), both CO_2_ and NH_3_ partial pressures can match experimental data within about 15% error. However, the model underestimates slightly the NH_3_ partial pressure and overestimated CO_2_ partial pressure at higher NH_3_ concentration and lower CO_2_ molality, which may be caused by the volatility of NH_3_. Nonetheless, under the conditions considered here, the largest difference between the calculation and experiments is about 12%.

#### 3.1.2 Validation of the thermodynamic model in liquid phase (solution speciation and SLE)

**Fig. 4:**
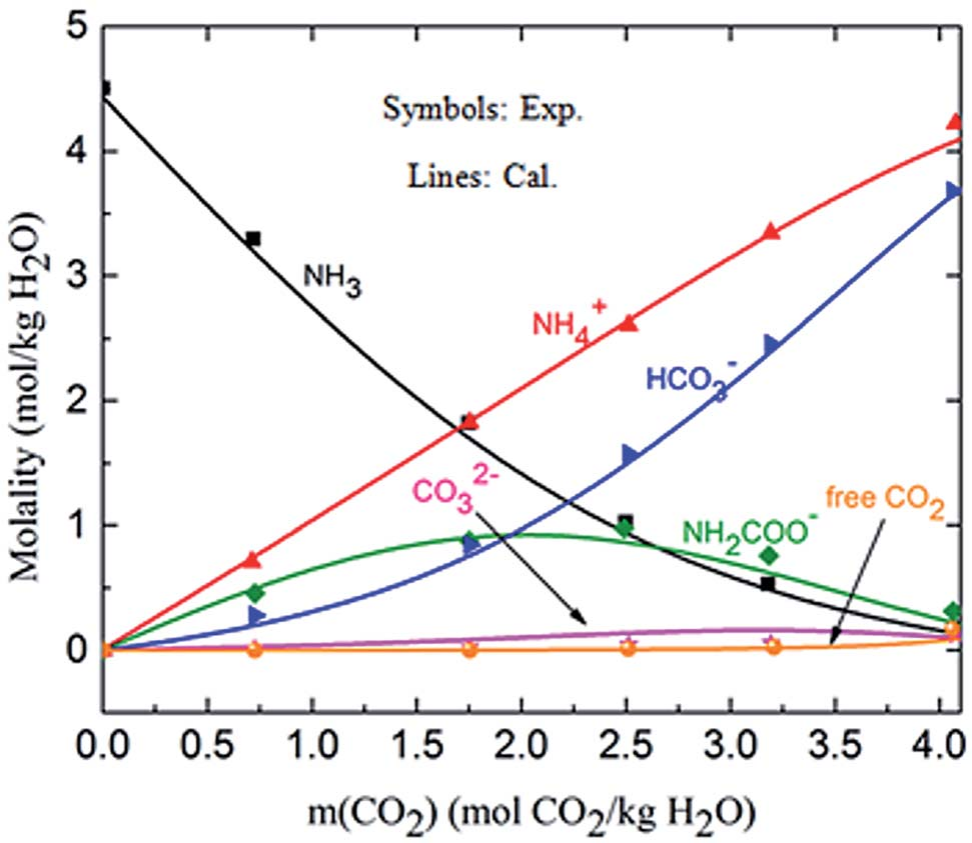
Comparison of the calculated solution speciation with experimental data^60^ at *T* = 60 °C and *m*(NH_3_) = 4.44 mol NH_3_/kg H_2_O.

Fig. 4 shows the calculated solution speciation and experimental results reported by Lichtfers and Rumpf.^60^ The corresponding conditions are *m*(NH_3_) = 4.44 mol kg^−1^ H_2_O and *T* = 60 °C. It concludes that the calculated results agree well with the experimental data within less than 6% error. The increase in carbamate molality is greater than for those of carbonate and bicarbonate in the presence of excess NH_3_ at the initial stage of absorption. The carbamate concentration reaches its maximum value at about *m*(CO_2_) = 2.2 mol CO_2_/kg H_2_O (CO_2_ loading = 0.5 mol CO_2_/mol NH_3_). However, at high CO_2_ molality (*m*(CO_2_) greater than 2.5 mol CO_2_/kg H_2_O) the bicarbonate is the dominant species. Meanwhile, the concentration of carbamate decreases.^61^

**Fig. 5:**
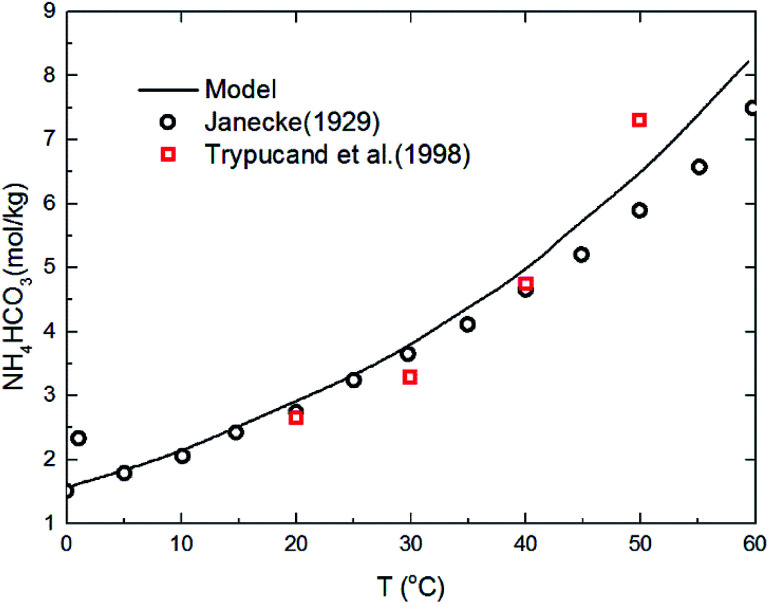
Comparison of NH_4_HCO_3_(s) solubility in ammonia solution at different temperatures between calculated values and data in literature.^62,63^

The deviation for NH_4_HCO_3_(s) solubility in ammonia solution between calculated and different literature values^62,63^ are shown in Fig. 5 at temperatures from 0 to 60 °C. The maximum and average deviations are 5% and 2%, respectively. The deviation of NH_4_HCO_3_(s) solubility between calculated and literature value at temperatures more than 40 °C is slightly higher than those at lower temperatures. However, considering the temperature ranges in the present study (from 2 to 40 °C), the relative deviation is less than 5% which confirms the accuracy of the thermodynamic model in this study.

#### 3.1.3 Validation of thermodynamic model by heat of absorption

**Fig. 6:**
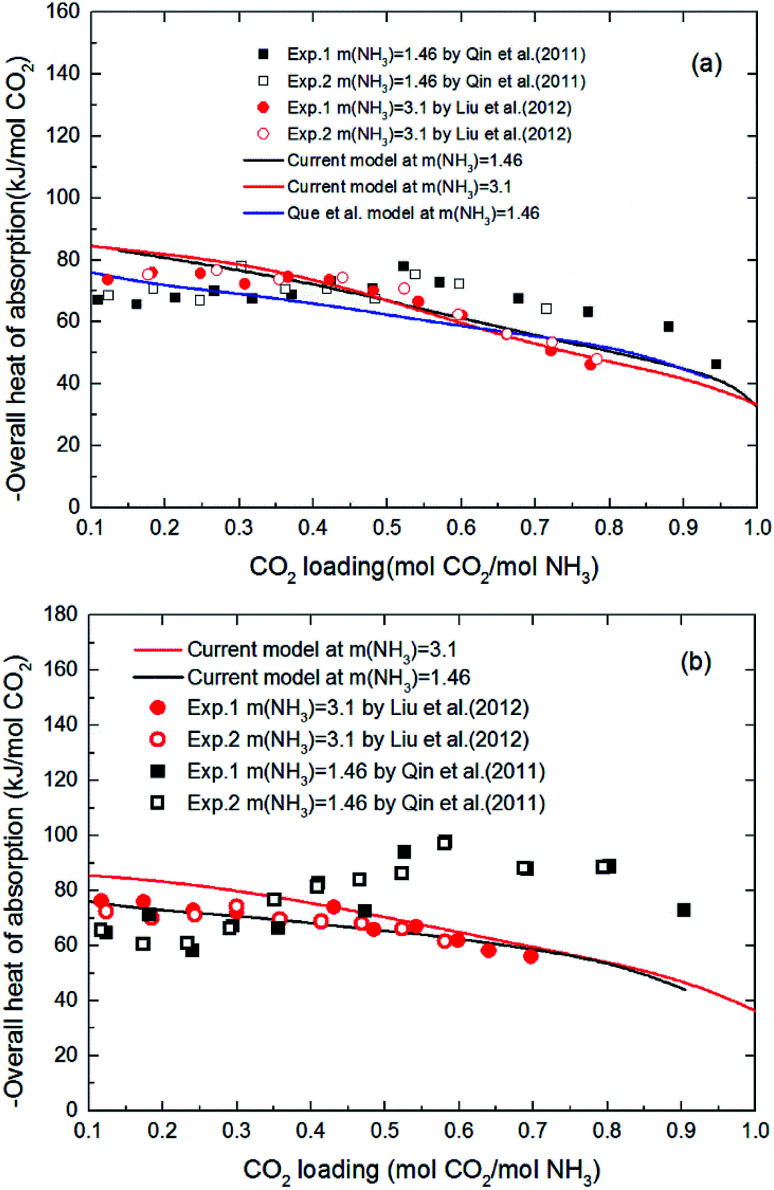
Comparison of the overall heat of CO_2_ absorption predicted by model with experimental data^20,21^ at different temperatures ((a) *T* = 40 °C, (b) *T* = 60 °C).

Fig. 6 shows the heat of CO_2_ absorption predicted by the model and the experimental data of Liu *et al.*^20^ and Qin *et al.*^21^ at different temperatures. In addition, another model from Que *et al.*^55^ is also cited in Fig. 6 for comparison. As we can see that all the model values and experimental data decrease with CO_2_ loading except Qin *et al.* Qin *et al.* found that the absorption heat of CO_2_ with NH_3_ at 40 °C and 60 °C decreases at first with increasing loading, but between 0.2 and 0.6 mol CO_2_/mol NH_3_ in loading it rapidly increases. When the loading is around 0.6 mol CO_2_/mol NH_3_, the absorption heat of CO_2_ with NH_3_ reaches a maximum (∼100 kJ mol^−1^ CO_2_ at 60 °C). The absorption heat then starts to decrease again. This trend is more pronounced at high temperature (60 °C). No theoretical justification for this strange trend is presented in their paper. However, according to all prior researchers' results, there is no reaction between CO_2_ and NH_3_ that should release a heat of absorption higher than 100 kJ mol^−1^ CO_2_.^20,36,55^ The estimated absorption heat of CO_2_ with NH_3_ using the speciation data of Mani *et al.*,^61^ measured by NMR, also gives a value of around 80 kJ mol^−1^ CO_2_. In addition, as CO_2_ is gradually absorbed, the concentration of ammonia in the solution decreases attenuating the reaction. The amount of heat released during the absorption process should be gradually reduced. So, the validity of the data obtained by Qin *et al.* needs further discussion. In general, the agreement between the current model values and Liu's experimental data, as well as agreement with the model values of Que *et al.* clearly support the model validity and accuracy. The subtle difference between the model values and experimental data may be caused by the activity change of species conjectured by Kim.^64^ The contribution to the heat of absorption from the liquid-phase nonideality is neglected in this study. It should be better to consider the heat from the liquid-phase nonideality in the model to examine Kim's guess in our future works. In addition, the modeling deviation may also be from the chemical equilibrium constants chosen from literature. As shown in Fig. 2(a), the chemical equilibrium constants chosen from different literature have some differences with each other and may cause a difference in the calculation of enthalpy change using eqn (8) (see Fig. 2(b)). The heat of CO_2_ absorption predicted by the model decreases from −81 to −37 kJ mol^−1^ with the CO_2_ loading increasing from 0.1 to 1 mol CO_2_/mol NH_3_. In addition, the current model results indicate that the overall heat of CO_2_ absorption does not change significantly with NH_3_ concentration. This implies that the reaction between NH_3_ and CO_2_ at different NH_3_ concentration has almost the same reaction products distribution.

### 3.2 Individual reaction contribution to the overall heat of CO_2_ absorption

**Fig. 7:**
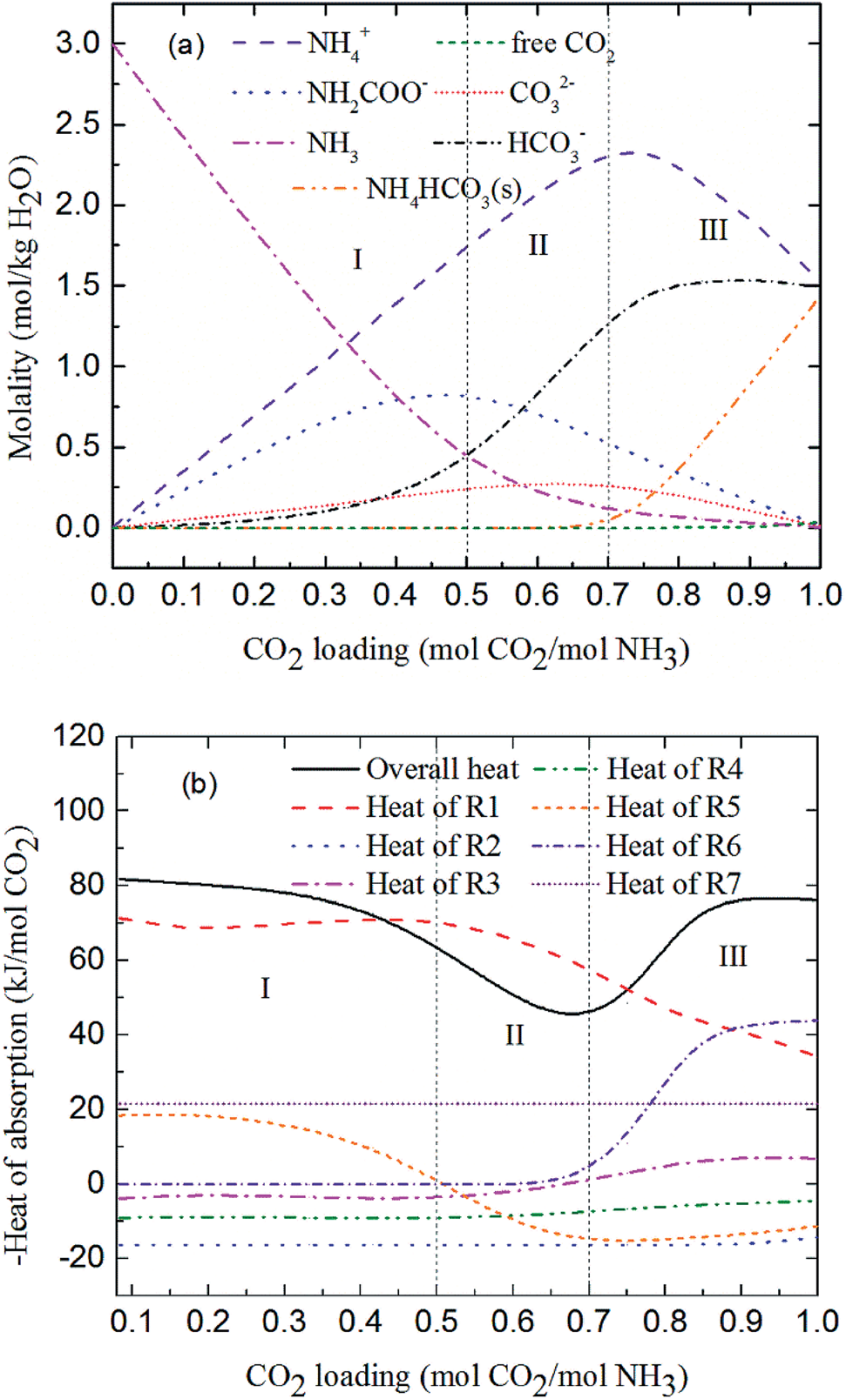
Prediction of (a) solution speciation change and (b) heat of CO_2_ absorption in the NH_3_–CO_2_–H_2_O system at *m*(NH_3_) = 3 mol kg^−1^ H_2_O and *T* = 2 °C.

Fig. 7 shows the predicted solution speciation and heat of CO_2_ absorption in the NH_3_–CO_2_–H_2_O system, respectively, all at *m*(NH_3_) = 3 mol kg^−1^ H_2_O and *T* = 2 °C. Because the formation of carbamate (NH_2_COO^−^) and NH_4_HCO_3_(s) significantly impact the heat of CO_2_ absorption, the whole absorption process is divided into three stages according to carbamate and NH_4_HCO_3_(s) formation, as shown in Fig. 7, *i.e.* Stage I: CO_2_ loading < 0.5 mol CO_2_/mol NH_3_; Stage II: 0.5 < CO_2_ loading < 0.7 mol CO_2_/mol NH_3_; and Stage III: CO_2_ loading > 0.7 mol CO_2_/mol NH_3_. They are discussed in detail in the following paragraphs.

At low CO_2_ loading (Stage I), there is an excess of free NH_3_, and carbamate is the main product in the solution *via* the forward reaction of carbamate formation (R5). For example, 0.333 mol CO_2_/mol NH_3_, 72% of CO_2_ converts to carbamate and only 12.5% and 15.4% converts to bicarbonate and carbonate, respectively. Fig. 7(b) shows that the overall heat of CO_2_ absorption first decreases and then increases rapidly with increasing CO_2_ loading. As explained above, (R5) moves forward to form carbamate with increasing CO_2_ loading in Stage I. In this stage, (R5) is an exothermic process (−Δ*H* of (R5) has a positive value) and thus releases heat.

As the absorption proceeds to Stage II, carbamate is decomposed *via* the backward reaction of carbamate formation (R5) to form bicarbonate, with 56.9% of CO_2_ turns into bicarbonate, 13.6% into carbonate, and 29.5% into carbamate at CO_2_ loading of 0.667 mol CO_2_/mol NH_3_. In this stage, (R5) moves backward with increasing CO_2_ loading. As shown in Fig. 7(b), (R5) is still the dominant reaction, but becomes an endothermic, thus reducing the overall heat of CO_2_ absorption (the overall process remaining exothermic).

Fig. 7(a) shows that for CO_2_ loading greater than 0.7 mol CO_2_/mol NH_3_ (Stage III), NH_4_HCO_3_(s) is gradually formed *via* the forward reaction of NH_4_HCO_3_(s) formation (R6) at 2 °C. The amount of bicarbonate produces by carbamate decomposition is equal to that consumes by solid formation, so the concentration of bicarbonate remains constant. The corresponding overall heat of CO_2_ absorption increases due to the heat release from the solid formation, which can be seen in Fig. 7(b). The overall heat of CO_2_ absorption is about −78 kJ mol^−1^ CO_2_ at CO_2_ loading of 1 mol CO_2_/mol NH_3_, which is similar to the initial stage of absorption. Now, NH_4_HCO_3_(s) formation (R6) contributes most to the overall heat of CO_2_ absorption. Water as a main reactant is continuously consumed by CO_2_ dissociation (R2), CO_3_^2−^ formation (R3) and NH_3_ protonation (R4), causing water ionization (R1) to move backward and to release heat in the entire absorption process. It is worth pointing out that the heat of CO_2_ physical absorption (R7) remains −21 kJ mol^−1^ CO_2_ or so in Fig. 7(b). This is because the Henry's law constant of CO_2_ physical absorption (R7) depends on temperature, and the physical absorption amount of CO_2_ increases linearly with increasing CO_2_ loading.^28^

**Fig. 8:**
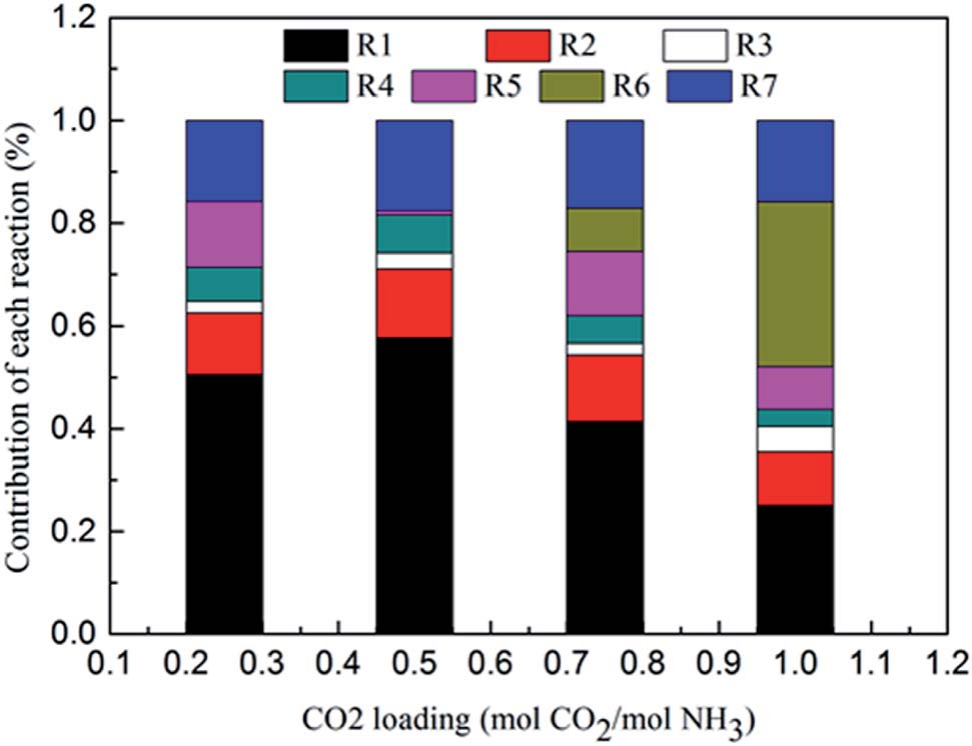
Contribution of each reaction to overall heat of CO_2_ absorption at *m*(NH_3_) = 3 mol kg^−1^ H_2_O and *T* = 2 °C.

Fig. 8 shows the contribution of each reaction to the overall heat of CO_2_ absorption at *m*(NH_3_) = 3 mol kg^−1^ H_2_O and *T* = 2 °C. The share of CO_3_^2−^ formation (R3) is very small due to the small amount of CO_3_^2−^ in the solution. The water dissociation (R1), CO_2_ dissociation (R2), carbamate formation (R5), and CO_2_ physical absorption (R7) are the main contributors to the overall heat of CO_2_ absorption at the initial phase (CO_2_ loading = 0.25 mol CO_2_/mol NH_3_). This is quite different from amine-based system. Kim *et al.*^27^ reported that the main contributors to the overall heat of CO_2_ absorption in MEA solution were carbamate and MEAH^+^ formation reactions. When CO_2_ loading is 0.5 mol CO_2_/mol NH_3_, the contribution of carbamate formation (R5) becomes minimum. This is because carbamate formation (R5) is at a tipping point from forward to backward reaction, when the extent of carbamate formation reaction (R5) is very weak. After the solids appear at CO_2_ loadings greater than 0.7 mol CO_2_/mol NH_3_, the NH_4_HCO_3_(s) formation (R6), water dissociation (R1), and CO_2_ physical absorption (R7) become the main contributors to the overall heat. The contribution of NH_4_HCO_3_(s) formation (R6) is 32% at a CO_2_ loading = 1 mol CO_2_/mol NH_3_.

**Fig. 9:**
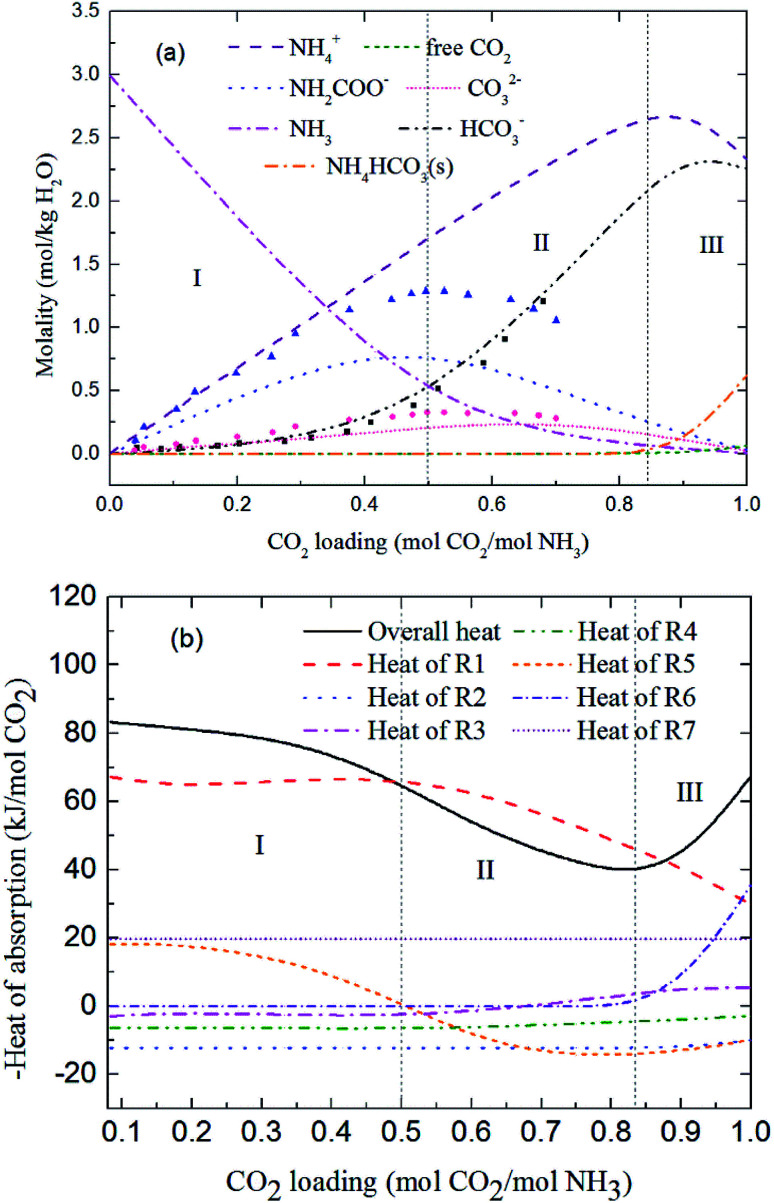
Predictions of (a) solution speciation change and (b) heat of CO_2_ absorption in the NH_3_–CO_2_–H_2_O system at *m*(NH_3_) = 3 mol kg^−1^ H_2_O and *T* = 15 °C: (■) HCO_3_^−^ (●) CO_3_^2−^ and (▲) NH_2_COO^−^ concentration in Jilvero *et al.*^31^ at *m*(NH_3_) = 3.5 mol kg^−1^ H_2_O and room temperature.

**Fig. 10:**
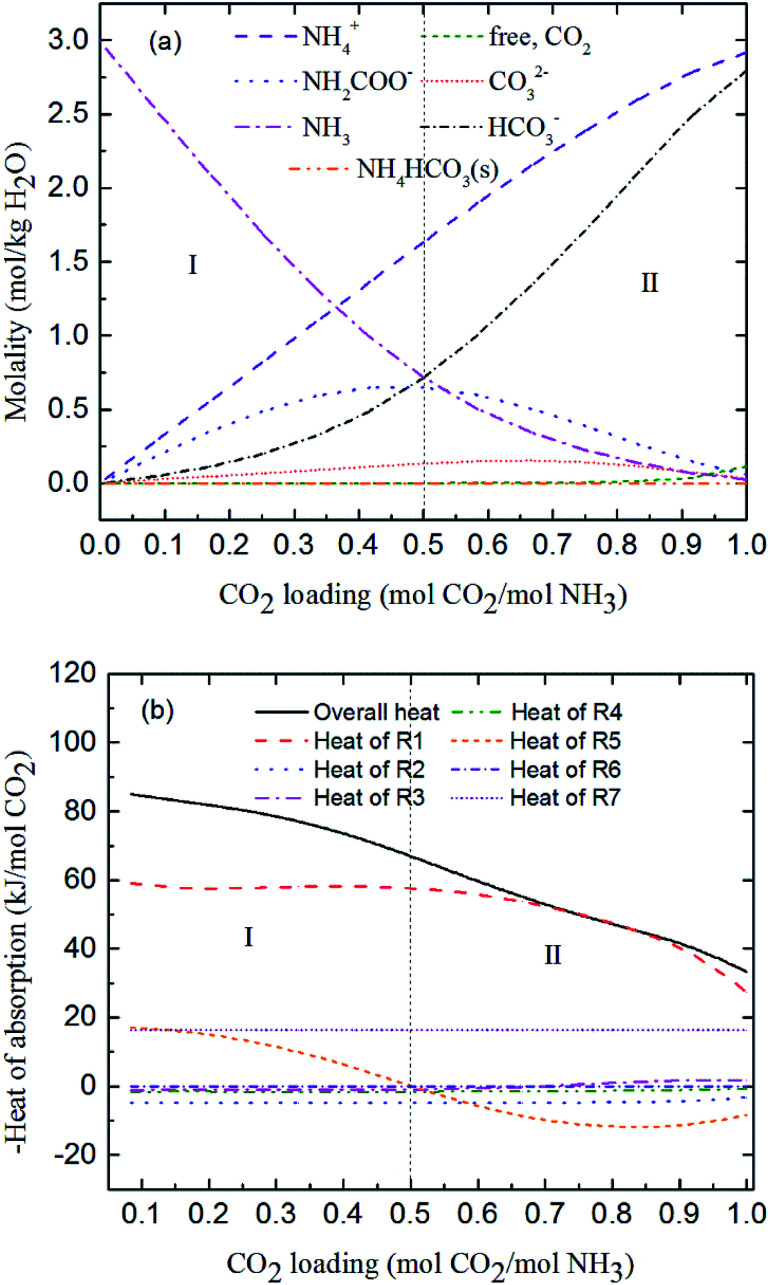
Predictions of (a) solution speciation change and (b) heat of CO_2_ absorption in the NH_3_–CO_2_–H_2_O system at *m*(NH_3_) = 3 mol kg^−1^ H_2_O and *T* = 40 °C.

Fig. 9 and 10 show the prediction of solution speciation change and heat of CO_2_ absorption in the NH_3_–CO_2_–H_2_O system at *T* = 15 °C and 40 °C, respectively. At *T* = 15 °C (Fig. 9), three stages, similar to the process at *T* = 2 °C (Fig. 7), are observed, but with a higher turning point of CO_2_ loading (moving from 0.7 at *T* = 2 °C to 0.85 mol CO_2_/mol NH_3_ at *T* = 15 °C). Additionally, speciation data reported by Jilvero *et al.*^31^ at *m*(NH_3_) = 3.5 mol kg^−1^ H_2_O and room temperature is also include in Fig. 9. The trend of the model results agree well with those of experimental data. However, the model values of NH_2_COO^−^ are distinctly lower than the experimental data. This is because the NH_3_ concentration in Jilvero *et al.* (*m*(NH_3_) = 3.5 mol kg^−1^ H_2_O) is higher than that in this study (*m*(NH_3_) = 3 mol kg^−1^ H_2_O). According to (R5), Higher NH_3_ concentration promotes the formation of NH_2_COO^−^, so the NH_2_COO^−^ concentration in Jilvero *et al.* is higher than our model results. When the absorption temperature increases further to 40 °C, only two stages can be seen in Fig. 10. The third stage caused mainly by the formation of NH_4_HCO_3_(s) disappears at higher temperature, as shown in Fig. 10.

### 3.3 Formation conditions of NH_4_HCO_3_(s) in CAP

**Fig. 11:**
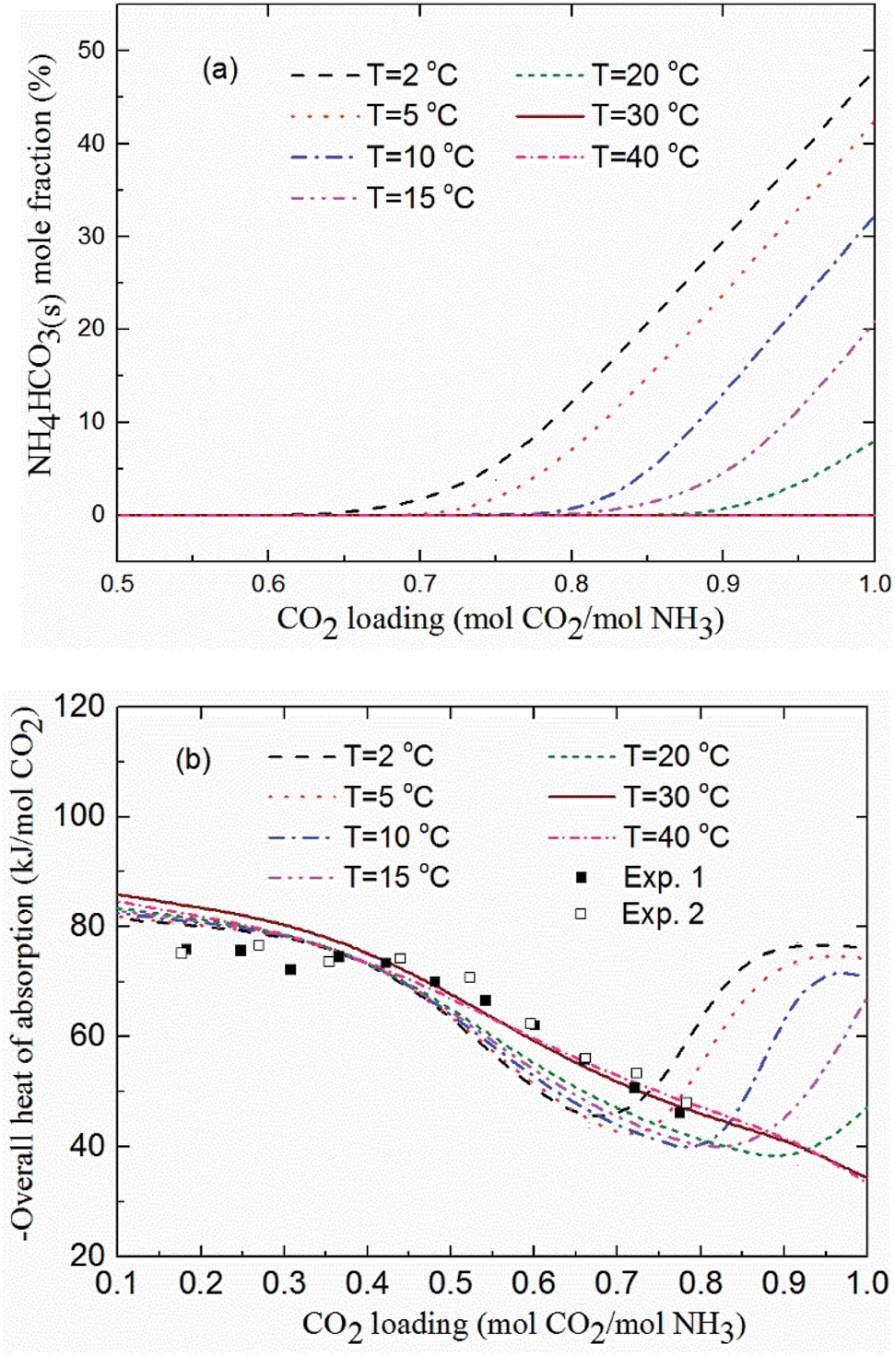
(a) NH_4_HCO_3_(s) mole fraction and (b) overall heat of CO_2_ absorption *vs.* CO_2_ loading at temperatures between 2 and 40 °C and *m*(NH_3_) = 3.1 mol kg^−1^ H_2_O (lines: model results, square points: experimental data^20^ at *T* = 40 °C and *m*(NH_3_) = 3.1 mol kg^−1^ H_2_O).

Fig. 11(a) shows the NH_4_HCO_3_(s) mole fraction in the solution at temperatures between 2 and 40 °C and for *m*(NH_3_) = 3.1 mol kg^−1^ H_2_O. The corresponding overall heat of CO_2_ absorption is shown in Fig. 11(b). As low temperature favors the formation of solid phase NH_4_HCO_3_(s),^46^ there is little solid formed (less than 8%) for temperatures over 20 °C. CO_2_ loading above 0.7 mol CO_2_/mol NH_3_ and temperatures less than 20 °C promotes NH_4_HCO_3_(s) precipitation, which can dramatically increase the heat of CO_2_ absorption. For instance, NH_4_HCO_3_(s) begins to form when CO_2_ loading is greater than 0.7 mol CO_2_/mol NH_3_ at *T* = 2 °C, and almost 50% of CO_2_ is converted to NH_4_HCO_3_(s) at CO_2_ loading = 1 mol CO_2_/mol NH_3_. The overall heat of absorption changes from −43.43 to −76.09 kJ mol^−1^ CO_2_ caused by NH_4_HCO_3_(s) formation at *T* = 2 °C (see Fig. 11(b)).

As shown in Fig. 11(b), the model results show a good agreement with the experimental data^20^ at *T* = 40 °C. The predicted average heat of absorption is about −74.4 kJ mol^−1^ CO_2_ at low CO_2_ loadings (0.2 mol CO_2_/mol NH_3_ < CO_2_ loading < 0.5 mol CO_2_/mol NH_3_). This is consistent with Liu *et al.*'s results (−74.8 kJ mol^−1^ CO_2_).^20^Fig. 11(b) also shows that temperature has almost no effect on the heat of CO_2_ absorption at low CO_2_ loadings (less than 0.5 mol CO_2_/mol NH_3_), which is consistent with the results from the model of Que and Chen.^55^ However, at high CO_2_ loadings (above 0.7 mol CO_2_/mol NH_3_), the decrease in temperature shows a negative effect on the overall heat of CO_2_ absorption. The overall heat of CO_2_ absorption at a CO_2_ loading of 0.9 mol CO_2_/mol NH_3_ are −77.1, −75.7, −73.3, −45.3 and −36.6 kJ mol^−1^ CO_2_ for temperatures of 2, 5, 10, 15 and 20 °C, respectively. This is likely the more amounts of NH_4_HCO_3_(s) at low temperature (see Fig. 11(a)) the more heat is released through NH_4_HCO_3_(s) formation reaction (R6). The formation of solid at low temperature greatly increases the overall heat of CO_2_ absorption. CO_2_ loading with the lowest absorption heat, 0.67, 0.75, 0.8, 0.83 and 0.92 mol CO_2_/mol NH_3_ at the corresponding temperature of 2, 5, 10, 15 and 20 °C are recommended in this study to avoid solid formation, which can, not only minimize the overall heat of CO_2_ absorption, but also mitigate fouling and blocking problems in stripper and tubes.

## 4. Conclusions

The following conclusions can be drawn from the results in this study.

(1) The contribution of individual reactions to the overall heat of CO_2_ absorption in chilled ammonia process (CAP) is modeling studied using Aspen Plus at temperatures between 2 and 40 °C. NH_4_HCO_3_(s) formation (R6) in low temperatures is dominant contributor for the overall heat of CO_2_ absorption at CO_2_ loading above 0.7 mol CO_2_/mol NH_3_.

(2) The overall heat of absorption in CAP first decreases and then increases quickly with increasing CO_2_ loading. The increase in heat of absorption is caused by the prominent heat release during the formation of NH_4_HCO_3_(s). The contribution of each individual reaction to overall heat of absorption can be controlled by adjusting the operation parameters, such as CO_2_ loading and temperature, to optimize overall heat of absorption in chilled NH_3_–CO_2_–H_2_O system.

(3) The main contributions to the heat of absorption of CO_2_ in CAP were from the water ionization (R1), NH_2_COO^−^ formation (R5), solid NH_4_HCO_3_(s) formation (R6) and CO_2_ dissolution (R7) which quite differed from the MEA system. With CO_2_ loading > 0.5 mol CO_2_/mol NH_3_, (R5) changes from an exothermic reaction to an endothermic reaction, which can significantly reduce the absorption heat of the system. When temperature is lower than 20 °C, the CO_2_ loading is recommended to be around 0.6–0.7 mol CO_2_/mol NH_3_, so that the overall absorption heat is at a low state (less than 60 kJ mol^−1^ CO_2_). On the other hand, under this CO_2_ loading, the generation of solid NH_4_HCO_3_(s) (R6) can be avoided.

(4) The overall heat of CO_2_ absorption does not change much with temperature at low CO_2_ loading (less than 0.5 mol CO_2_/mol NH_3_). With a high CO_2_ loading (more than 0.7 mol CO_2_/mol NH_3_), the decrease in temperature has a negative effect on the heat of absorption.

(5) It should be better to consider the contributions from the liquid-phase nonideality in the model and the effect of other acid gases on the overall absorption heat by chilled ammonia process in our future works (*e.g.* the overall heat of absorption in chilled NH_3_–CO_2_–SO_2_–H_2_O system).

## Conflicts of interest

There are no conflicts to declare.

## Nomenclature

K
: Equilibrium constant
*k*_H_
: Henry's law constant, Pa
T
: Temperature, K
n
: Number of moles
H
: Enthalpy, J mol^−1^
R
: Gas constant, J mol^−1^ K^−1^
E
: Extent and direction of each reaction
f
: Fugacity

### Subscripts

*k*
: Reaction number
*m*
: Molality basis
*x*
: Mole fraction basis
*i*
: Key species i
tot: Total amount of CO_2_
abs: Absorption

### Greek letters

Δ: Change
∑: Summation
*φ*
: Fugacity coefficient
*γ*
: Activity coefficient

### Superscript

F: Final state
I: Initial state

## Supplementary Material
